# Kimberlite eruptions driven by slab flux and subduction angle

**DOI:** 10.1038/s41598-023-36250-w

**Published:** 2023-06-06

**Authors:** Ben R. Mather, R. Dietmar Müller, Christopher P. Alfonso, Maria Seton, Nicky M. Wright

**Affiliations:** EarthByte Group, School of Geosciences, The University of Sydney, Sydney, 2006 Australia

**Keywords:** Geodynamics, Tectonics

## Abstract

Kimberlites are sourced from thermochemical upwellings which can transport diamonds to the surface of the crust. The majority of kimberlites preserved at the Earth’s surface erupted between 250 and 50 million years ago, and have been attributed to changes in plate velocity or mantle plumes. However, these mechanisms fail to explain the presence of strong subduction signatures observed in some Cretaceous kimberlites. This raises the question whether there is a subduction process that unifies our understanding of the timing of kimberlite eruptions. We develop a novel formulation for calculating subduction angle based on trench migration, convergence rate, slab thickness and density to connect the influx of slab material into the mantle with the timing of kimberlite eruptions. We find that subduction angles combined with peaks in slab flux predict pulses of kimberlite eruptions. High rates of subducting slab material trigger mantle return flow that stimulates fertile reservoirs in the mantle. These convective instabilities transport slab-influenced melt to the surface at a distance inbound from the trench corresponding to the subduction angle. Our deep-time slab dip formulation has numerous potential applications including modelling the deep carbon and water cycles, and an improved understanding of subduction-related mineral deposits.

## Introduction

Kimberlites are mafic volcanic rocks erupted from the Earth’s mantle and are the host rocks of most diamonds^1^. Kimberlites occur on every craton and have sporadically been emplaced since 3 Ga^2^, yet the greatest number of kimberlite eruptions preserved on Earth today formed during the last 250 to 50 million years, primarily in Africa and North America^3^. While the distribution of kimberlites has been associated with the edges of large low-shear-wave-velocity provinces (LLSVPs)^4^, and changes in angular plate velocity^3^, these do not explain the frequency of kimberlite eruptions nor enriched radiogenic isotope signatures indicating a subducted slab component in some Cretaceous kimberlite populations^1,5^. The steep subduction of oceanic lithosphere into the mantle has been proposed to drive strong mantle return flow and pulses of magmatism^6^. Yet, despite the theoretical connection between volcanic eruptions and high rates of slab flux^7^, difficulties in estimating the volume and subduction angle of oceanic lithosphere being recycled at ancient subduction zones has thwarted any correlation with kimberlite eruptions. Previous attempts to characterise the dip angle of subducting slabs applied multivariate analysis of subduction zone characteristics to search for correlations among key parameters^8–14^. However, these approaches are mostly useful for reproducing the present-day slab dip and have limited application to subduction zones reconstructed through deep geological time. Here, using a recent tectonic plate reconstruction model^15^ and models of plate cooling^16–18^, we revisit the estimation of slab dip from simple plate kinematic parameters that characterise most subduction zones across the globe to explore the potential role of steeply subducting slabs in controlling kimberlite eruptions in Africa and North America.

**Figure 1:**
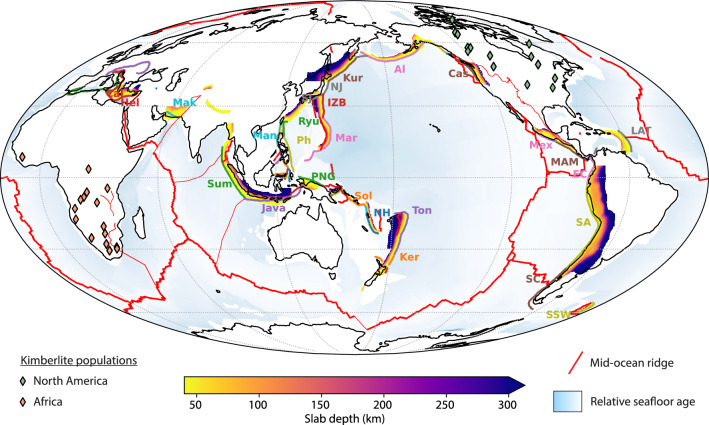
Depths of subducting slabs obtained from the Slab2 model^19^ overlain with kimberlites erupted within the last 250 million years^20^. Trench segments have the following abbreviations in (i) Oceania: Ton, Tonga; Ker, Kermadec; NH, New Hebrides; Sol, Solomon; (ii) Southeast Asia: PNG, Papua New Guinea; Sum, Sumatra; Mar, Marianas; IZB, Izu-Bonin; Ryu, Ryukyu; Man, Manila; Ph, Philippine; (iii) Asia: Mak, Makran; SJ, South Japan; NJ, North Japan; Kur, Kurile; (iv) Europe: Hel, Helenic; Cal, Calabria; (v) North America: Al, Aleutians; Cas, Cascades; (vi) Central America: Mex, Mexico; MAM, Middle America; LAT, Lesser Antilles; (vii) South America: EC, Ecuador; SA, South America; SC, South Chile; SSW, South Sandwich. Approximate trench locations, boundary types, names, and corresponding abbreviations are listed in Table S1. White areas indicates regions of non-oceanic crust; thick red lines indicate mid-ocean ridges; thin red lines indicate transform boundaries. Map was generated using Cartopy^21^.

## Relationships between slab dip, plate kinematics and rheology

The downward pull of slabs into the Earth’s mantle is the largest driving force in plate tectonics^22^, where the bending of the subducting plate plays an important role in modulating the amount of slab pull which is transferred to the surface to drive plate motion^23^. Since the 1980s^8^, multiple studies have explored correlations between subduction angle and a number of parameters, including the duration of subduction, convergence rate, nature of the over-riding plate, trench length and others^8–14^. A recent study returned to a multivariate regression and reinforced the long-held view that slab dip is influenced by subduction duration and, to a lesser extent, the age of the down-going plate and whether the overriding plate is continental or oceanic in nature^13^. While these parameters are useful for approximating the present-day slab dip, the demarcation of subduction zone segments are subjective and time-dependent, which poses a difficulty when attempting to cast these slab dip relationships back through deep geological time with constantly evolving subduction boundaries. For example, data on the duration or re-initiation of subduction is increasingly sparse with age and plate reorganisations render subduction longevity difficult to quantify. Similarly, the subdivision of slab segments through deep time is highly subjective and a break in subduction zone topology may result in vastly different slab dip estimates. To overcome these challenges, we explore slab dip correlations with a multivariate analysis of plate rheology and kinematic parameters that are less ambiguous through deep geological time due to the optimisation of plate models for no-net rotation^24^. By taking this approach, the estimation of slab dip can be applied to evolving subduction zones stretching back as far as a given plate reconstruction will allow.

**Figure 2:**
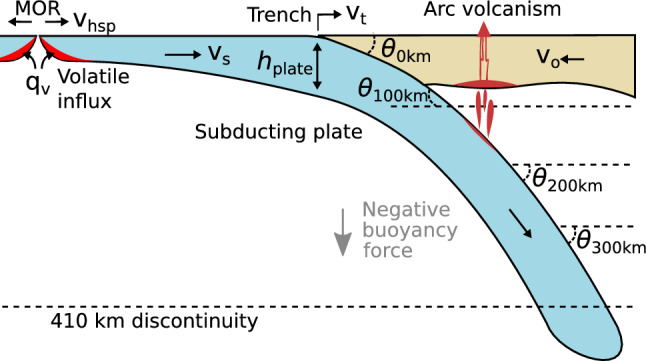
Schematic representation of slab dips and subduction parameters. The convergence rate, $v_c$, is the summation of the velocity of the subducting plate, $v_s$, and the overriding plate velocity, $v_o$. We assume the velocity of the overriding plate and the trench are equal and opposite ($v_o = -v_t$). $v_s$ and $v_o$ are positive towards the trench and each vector is orthogonal to the trench. $v_{\textrm{hsp}}$ is the half-spreading rate at mid-ocean ridges, which is proportional to the rate of volatile influx ($q_v$) into the plate, and $\theta$ is the dip angle of the subducting slabs calculated at different intervals.

We extract present-day slab dip data from the Slab2 model^19^, which estimates the depth and geometry of subducting slabs across the globe from earthquake depths and tomography models (Fig. 1), and combine those with close to present-day plate kinematic properties obtained from a recent plate reconstruction model^25^ using pyGPlates^26^. The dip angle, $\theta$, of the down-going slab is taken at multiple depth intervals inboard from the trench (Fig. 2). The average dip angle, $\theta _{\textrm{av}}$ is simply the arithmetic mean of all depth intervals resolved by the Slab2 model orthogonal to the trench,

1 $$\begin{aligned} \theta _{\textrm{av}} = \frac{1}{n} \sum _{d=0}^{n} \theta _d \end{aligned}$$

where *d* is the depth interval and *n* is the total number of depth intervals that may be resolved by the Slab2 model. The entire workflow to calculate the dip angle from the Slab2 model as well as plate rheology and kinematic parameters of the subducting plate are openly available on GitHub (https://github.com/brmather/Slab-Dip). The most statistically significant combinations of parameters that are sensitive to the present-day dip angle of subducting slabs are described in following sections.

### Slab flux

**Figure 3:**
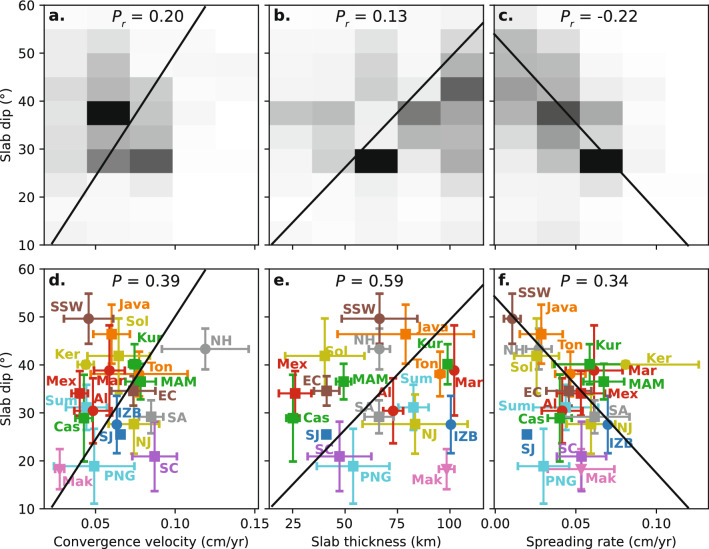
Correlations between slab dip and convergence velocity, slab thickness, and spreading rate. 2D histograms illustrate the probability density between each parameter with slab dip for all subduction zone segments tessellated at 0.5 degree increments (**a**–**c**); subduction zone segments are grouped for each subduction zone with whiskers indicating one standard deviation from the mean (**d**–**f**). $P_r$ is the Pearson correlation coefficient value and *P* is the p-value.

Previous studies on the multivariate analysis of subduction coefficients did not find a statistically significant relationship between slab dip and the age of subducting oceanic lithosphere^8,9,13^. However, we find a significant relationship where the slab dip is proportional to the convergence velocity, $v_c$ ($P=0.39$), and the thickness of the down-going plate, $h_{\textrm{plate}}$ ($P=0.59$) (Fig. 3). Plate thickness was predicted from the thermal evolution of oceanic lithosphere,

2 $$\begin{aligned} \theta _{\textrm{av}} \propto v_{c} \sqrt{\kappa t} \end{aligned}$$

where $\sqrt{\kappa t}$ derives from models of plate cooling^16,27^. These models describe the thickening of the thermal boundary layer as a function of the age of seafloor, which approaches a maximum thickness around 80 Myr for a constant thermal diffusivity coefficient of $\kappa = 1$ mm$^2$/s. We employ a plate model of oceanic lithosphere cooling, which has been demonstrated to produce an optimal fit to depth and heat flow data, to calculate the thickness of oceanic lithosphere being recycled at trenches across the Earth^16^. The product of the plate thickness, $h_{\textrm{plate}}$, and the convergence velocity, $v_c$, integrated along a trench segment gives the volumetric rate of lithospheric recycling, or “slab flux”. We sample seafloor age grids at trench boundaries to calculate the thickness of subducting oceanic lithosphere.

### Rollback

The second significant parameter to consider is the rate of trench advance or retreat. Slab rollback is widely regarded to lead to low-angle subduction and slab stagnation at the upper and lower mantle boundary^12,28,29^, which partly explains why lower subduction angles are more widely observed at subduction zones between two oceanic plates, where trench retreat is more common, than at ocean–continent subduction zones^10^. The rate of trench migration, $v_t$, is calculated relative to the mantle reference frame and can be compared to the convergence velocity, $v_c$, to characterise different subduction dynamics:

- If $v_c = -v_t$ the entire convergence rate is partitioned to rollback;
- If $v_t = 0$ the trench is stationary and the velocity of the subducting plate is equal to the convergence rate ($v_s = v_c$);
- If $v_t > 0$ the trench is advancing in the direction of subduction.

### Volatile enrichment

A third parameter we considered is the volatile enrichment of the subducting plate. An increased abundance of volatile components, such as water and carbon, enhances the coupling between the subducting plate to the overriding plate^30^. The source of volatile enrichment occurs at mid-ocean ridges, where volatile-bearing melt circulates through channels in newly-forming oceanic lithosphere^31^. The sequestration rate of volatiles within oceanic lithosphere, $q_v$, is proportional to the seafloor spreading rate, $v_{\textrm{hsp}}$^31^. We sample seafloor spreading rate grids, generated using workflows in^32^, at trench boundaries in the same way that we interpolate age grids to calculate plate thickness, $h_{\textrm{plate}}$. We find that $v_{\textrm{hsp}}$ exhibits a strong negative correlation with slab dip ($P=0.34$, Fig. 3c,f). Volatiles are also added to the plate by other processes, such as hydrothermal alteration of the upper crust^33^, sepertinisation during ultraslow spreading^34^, and during bending and cracking of the plate before entering the subduction zone^35^, however, the spreading rate relationship we outline above constitutes the most general source of volatiles subducted at most trenches globally.

### Buoyancy

A fourth parameter we considered was the mean density of the subducting plate. Lithospheric buoyancy can be estimated from plate age, thermal structure, crustal thickness, and depletion^36^. The crust is the main source of positive buoyancy in oceanic lithosphere due to its relatively low density ($\sim$ 2900 kg/m$^3$)^37^. Assuming modern mantle temperature conditions, 10–20 Myr old lithosphere is negatively buoyant^36^ and becomes more negatively buoyant with age. A 60 Myr segment of oceanic lithosphere is approximately 79.4 km thick^16^, 7 km of which is crust, which equates to a mean density of $\rho _{\textrm{av}} = 3278$ kg/m$^3$. While the buoyancy of oceanic plates may not be sufficient to initiate subduction alone^38^, lateral changes in the density structure of the down-going plate change the buoyancy force in established subduction zones which may affect slab dip. Such positive buoyancy anomalies are associated with oceanic plateaus which often congest subduction zones^39^ or lead to flat slab subduction^40^.

## Estimating slab dip

**Figure 4:**
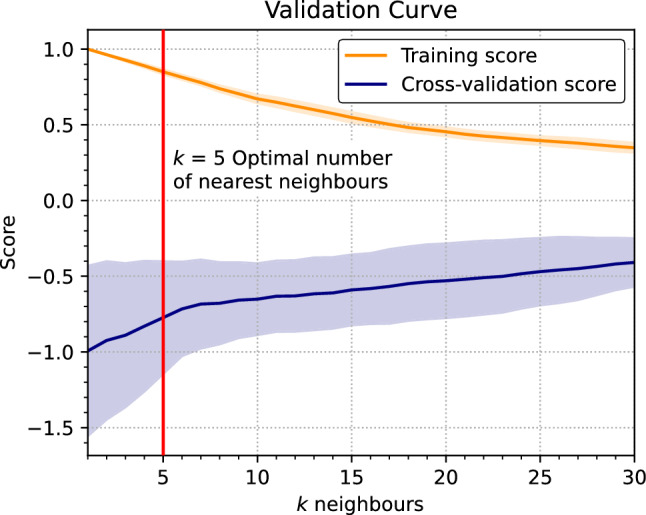
Cross-validation score and training score ($R^2$ value) for the nearest-neighbours regression algorithm to predict the dip angle of subducting slabs. The algorithm is tested using increasing numbers of nearest neighbours (*k*) used in the calculation from Eq.  3. Shaded regions indicate one standard deviation from the mean (solid lines). Cross validation score is shown as negative number for visual clarity to compare the optimal trade-off with the training score.

Taking all of these rheological and kinematic relationships into consideration for present-day subduction zones, we applied a nearest-neighbour regression to predict the dip angle, $\theta _{\textrm{av}}$, of subducting slabs. This regression implements a k-d tree to efficiently lookup *k* number of neighbours with the shortest euclidean distance from the training dataset $X_{\textrm{train}}$ to the test dataset $X_{\textrm{test}}$, and takes the weighted mean of corresponding slab dips to predict the test slab dip.

3 $$\begin{aligned} \theta _{\textrm{test}} = \frac{\sum _{k=1}^n d_k \theta _{\textrm{train}}(X_{\textrm{train,k}})}{\sum _{k=1}^n d_k} \end{aligned}$$

where $d_k$ is the euclidean distance between the training and test data for *k* nearest neighbours, $d_k = \Vert X_{\textrm{train}} - X_{\textrm{test}} \Vert _k$. Using a subset of the present-day configuration of subduction boundaries and slab dips obtained from the Slab2 model as the test dataset, we compare the performance of the slab dip prediction against the training dataset (Fig. 4). The training score ($R^2$ value) measures the closeness of fit between the training and test dataset which is a maximum of 1 for $k=1$. This is because there is an exact match for the test dataset from the training dataset at the present day. As *k* gets larger, more neighbours are incorporated within the average which reduces the $R^2$ value and the cross-validation score. The combination of these two metrics evaluate the estimator performance such that the problem is not over-fit or under-fit. We opt for $k=5$ where there is an optimal trade-off between the cross validation and training scores.

We have developed a flexible object-oriented Python package to estimate slab dip based on present-day plate rheology and kinematic parameters (https://github.com/brmather/Slab-Dip). The default regression algorithm and training dataset have been described above, however, the user can also define their own training dataset and any regression algorithm bundled within the scikit-learn Python package to create a bespoke slab dip estimator. The GitHub repository has several examples provided as Jupyter notebooks for the user as well as installation instructions. The nearest neighbours regression we have chosen is general and establishes a robust link between present-day properties of subducting plates with those through deep time. While it may not be applicable in specific geodynamic contexts that differ from those operating at the present day, it encapsulates some of the primary drivers of subduction that are predicted from plate tectonic theory such as the relationship between seafloor age, slab thickness, trench migration, and convergence velocity. This recognises that the slab suction force, associated with older dense slabs which sink sharply into the mantle, drives the velocity of the tectonic plates at the surface of the Earth, and an increase in the amount of rollback leads to a flattening of the down-going slab.

It is important to note that the relationship we have formulated predicts the slab dip down to the maximum depth resolved by the Slab2 model. The trajectory of the down-going slab into the mantle may deviate from the predicted slab depths as it encounters viscosity contrasts in the mantle which sometimes leads to slab stagnation^41,42^ and potentially slab anchoring^43^. These dynamics are not captured by our regression which may preclude its application to specific subduction zones over a nominal time range. Nevertheless, our slab dip formulation holds for most subduction zones globally and poses an advantage that it may be applied back through deep geological time using tectonic plate reconstructions to predict the trajectory of subducting slabs into the mantle. This can be used to estimate the distance between trenches and volcanic arcs, the incidence of flat slab subduction, and the recycling of oceanic lithosphere into deeper portions of the mantle.

## Subduction controls on kimberlite eruption

**Figure 5:**
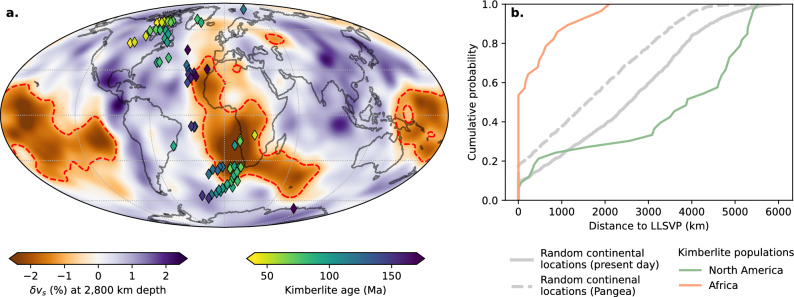
Spatio-temporal association between kimberlite eruptions and LLSVP boundaries. (**a**) Shear wave velocity anomaly from the SMEAN2 tomography model^54^ overlain with the spatial distribution of kimberlites (diamonds) reconstructed to their eruption age. We define LLSVP boundaries as the 1% slow contour of the 2800 km depth slice from the velocity variation which is represented by the red dashed line. Present-day coastlines are added for reference. Map was generated using Cartopy^21^. (**b**) Cumulative density function of the distance between LLSVP boundaries and kimberlite populations in Africa and North America with random continental locations at the present day and reconstructed to 170 Ma when the Pangea supercontinent was assembled. African kimberlites hold a statistically significant relationship to LLSVP boundaries while North American kimberlites do not.

**Figure 6:**
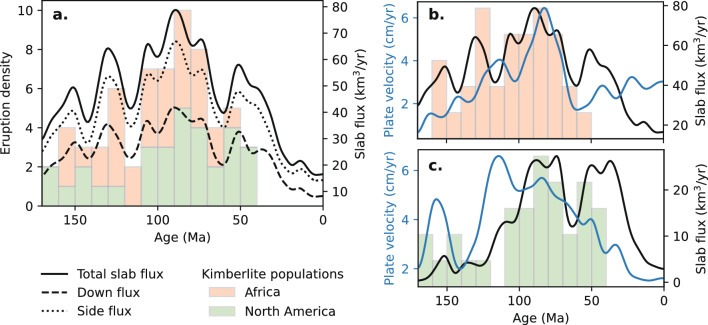
Relationship between kimberlite eruption density, slab flux, and plate velocity over 170 million years. (**a**) Pulses in combined kimberlite eruptions correlate with periods of high slab flux of the east-dipping Farallon plate along the western margin of North America. The low dip angle of subduction (30–35$^\circ$) predicted from our analysis is indicated by a strong sideways component of slab flux. (**b**) the first peak in African kimberlite eruptions is correlated with high slab flux between 120 and 130 Ma as Pangea dispersed; the second peak at 80–90 Ma is more likely to be explained by an increase in African plate velocity^3^ than slab flux due to the the increased distance between the Americas and Africa from the opening of the South Atlantic Ocean. (**c**) Pulses in North American kimberlite eruptions is closely correlated to pulses in slab flux integrated along east-dipping subduction zones within 2500 km radius of the nearest kimberlite eruption in North America with no correlation observed with plate velocity.

Kimberlites are volcanic rocks which ascend rapidly from the mantle and are emplaced in cratons worldwide^2^. Kimberlite eruptions have been associated with mantle upwellings from the LLSVP^4^ and extensional tectonics associated with lithospheric unloading^44^ or changes in plate velocity^3^. However, these mechanisms do not explain the strong subducted slab signatures observed in Cretaceous kimberlites in Africa, Brazil, and North America from increased strontium-isotope ratios^1,5^. While African kimberlites exhibit a statistically significant relationship with the distance to LLSVPs, kimberlites in North America hold no such relation (Fig. 5). In contrast, kimberlite eruptions in North America have been linked to flat subduction of the Farallon plate during the Laramide Orogeny^45^. Here, magma may have been generated from water-fluxed decompression melting of the mantle transition zone^46^ and transported upwards by subduction-induced mantle return flow^47^. High slab flux has been previously connected to volcanic eruption frequency, where mantle upwellings are driven by large volumes of oceanic lithosphere being subducted into the mantle^48^. Subduction-driven mantle upwellings have been linked to the formation of the 260 Ma Emeishan large igneous province in SW China^49^ and Cenozoic volcanism in NE China^50^. To reconcile the role of subduction in generating distinct kimberlite populations in Africa and North America, we separated the vertical and horizontal components of slab flux using our formulation of slab dip in the previous section, and reconstructed global subduction boundaries using pyGPlates^26^. We used a 170 Myr plate model, modified from^15^, with significantly improved subduction zone boundaries along the western margin of North America, and improved resolution of the Caribbean plate^51^ (refer to Methods). The locations of kimberlites $\le$ 170 Ma were reconstructed back to their time of eruption, using a compilation of kimberlites with eruption ages^52^ that we resampled to a 3-times refined icosohedral mesh^53^ to avoid duplication and geographic sampling bias. We then separated the kimberlites into (i) North America and (ii) Africa populations. Combined, these kimberlite populations constitute 91% of the global kimberlite dataset which have erupted within the last 250–50 million years termed the “kimberlite bloom”^3^. In comparing the timing of kimberlite eruptions with rates of slab flux, we only consider trenches where subduction is in the direction of kimberlite populations. We find a strong correlation between high slab flux along the western margin of North and Central America, associated with the subduction of the Farallon plate, with both African and North American kimberlite populations (Fig. 6a). The low subduction angle (30–35$^\circ$) predicted from our slab dip analysis on reconstructed subduction zones indicates that slabs would extend more than 1,000 km from the trench before intersecting the 660 km mantle transition zone, and may penetrate deeper into the lower mantle. Rapid subduction of slab material can produce mantle return flow^35^, from which mantle upwellings may drive kimberlite eruptions. In the following sections we explore how subduction-induced mantle return flow from high rates of slab flux may be connected with kimberlite eruptions within Africa and North America.

### African kimberlites

**Figure 7:**
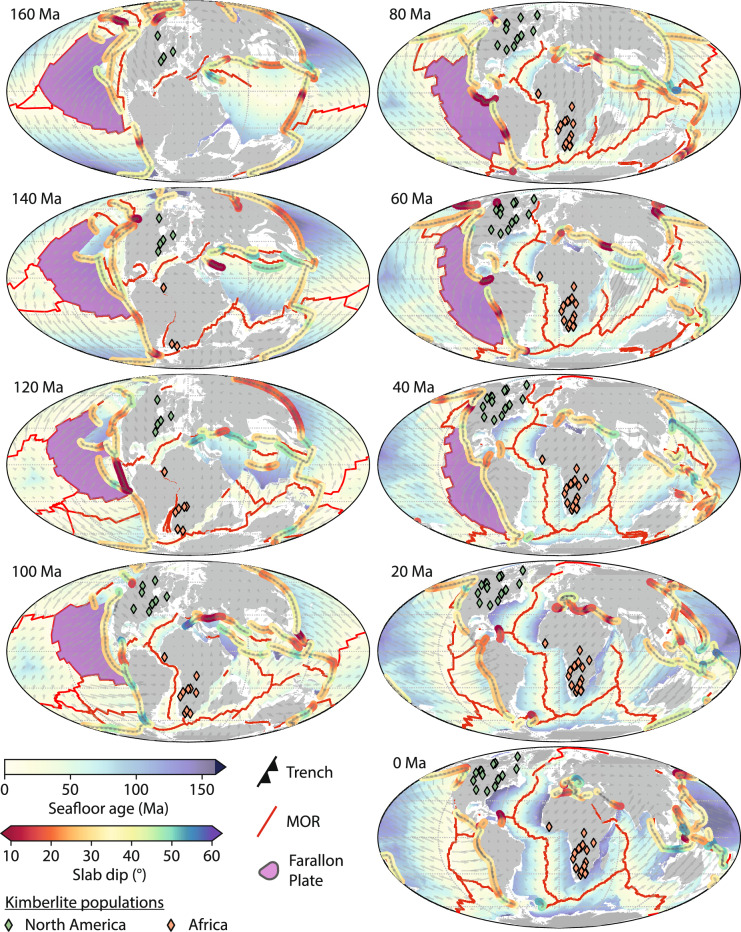
Evolution of slab dip angle at global subduction zones from 160 Ma to the present day overlain with the spatial distribution of kimberlite eruptions. A large peak in slab flux and kimberlite eruption density occurs at 120 Ma during the breakup of the Gondwana supercontinent, and a second (smaller) peak at 80 Ma associated with the removal of the flat Farallon slab from the base of overriding continental lithosphere in North America. White regions indicate non-oceanic crust, grey regions indicate present-day coastlines. Maps were generated using Cartopy^21^. A full timeseries from 170 Ma to 0 Ma is available as an animation in Movie S1.

A strong correlation exists between slab flux and the frequency of kimberlite eruptions during the peak in African kimberlite eruptions between 120 and 130 Ma (Fig. 6b). Subduction persisted along the western margin of the Pangea supercontinent during its assembly until rifting commenced between Africa and South America at approximately 120 Ma (Fig. 7). From 160 to 120 Ma, two peaks in kimberlite eruptions correlate with pulses of high slab flux from the subducting Farallon plate beneath the Americas which plunged 30–35 $^\circ$ into the mantle. The larger peak in kimberlite eruptions at 120 Ma corresponds to a slab flux of 60 km$^3$/yr. A second peak in African kimberlite eruptions occurred between 80 and 90 Ma, which correlates with a second pulse in slab flux (up to 80 km$^3$/yr) and a maxima in plate velocity (6 cm/yr) as the rate of seafloor spreading increases between Africa and South America (Fig. 6b). While it has been shown that mantle plumes associated with the LLSVP have eroded a significant proportion of cratonic lithosphere in Africa^55^, and may be associated with some kimberlite eruptions^4^, this does not explain the subduction signatures in kimberlites or the timing of their eruption.

We propose that a reservoir of recycled slabs occupy the mantle from pervasive subduction during the assembly of Pangea, which results in dehydration melting of the overlying mantle as water entrained with cold slabs is released^56^. Then, as Pangea begins to break-up, the rapid subduction of slab material at a low angle drives mantle return flow from this fertile mantle reservoir to drive kimberlite eruptions. Since subducting slabs influence the deep mantle structure, potentially triggering enhanced plume flux at the edges of the African LLSVP^7^, this could accelerate the delivery of slab-influenced melt from the uppermost lower mantle to the surface corresponding to an enhanced frequency of kimberlite eruptions at 120 Ma. This may be similar to a process contributing to the formation of the 260 Ma Emeishan large igneous province, where recycled Palaeo-Tethys Ocean beneath SW China has been proposed to induce large-scale mantle upwellings from 410-660 km depths^49^. The second pulse in kimberlite eruptions at 80–90 Ma cannot readily be linked to slab flux due to the vast distance from the nearest subduction zone following the opening of the South Atlantic Ocean (Fig. 7. Instead, an increase in African plate velocity would expose more cratonic lithosphere to mantle upwellings connected to the LLSVP, thereby increasing the kimberlite eruption frequency^3,4^ (Fig. 6b).

### North American kimberlites

**Figure 8:**
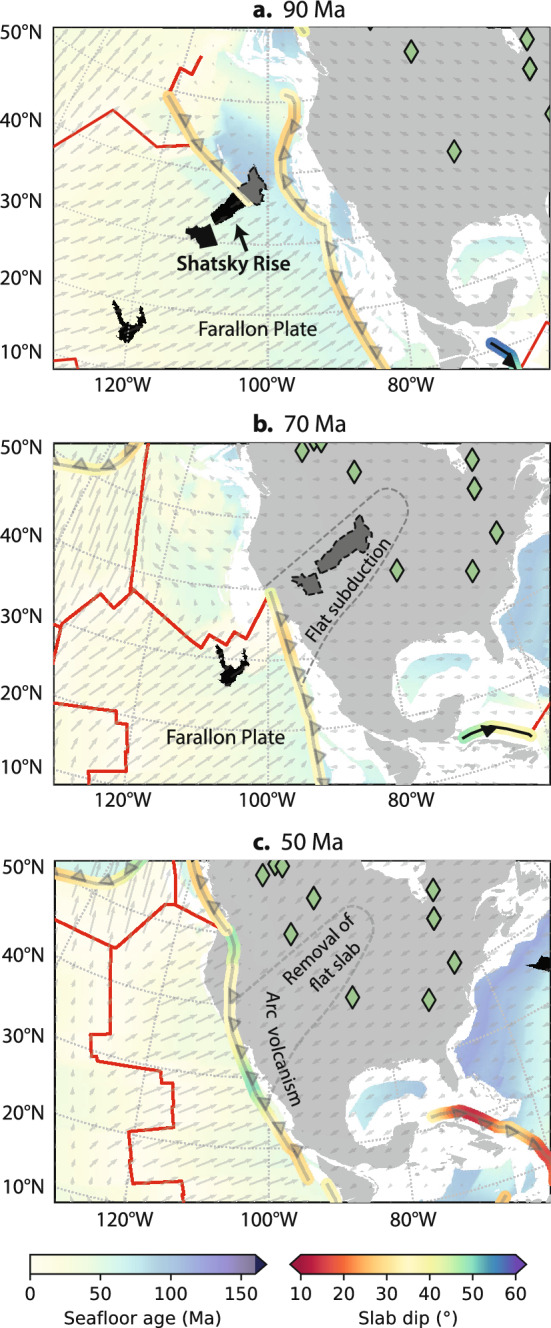
Evolution of flat slab subduction along North America. (**a**) Flat slab subduction of the Farallon Plate is caused by the Shatsky Rise which enters the trench at 95 Ma. (**b**) Much of the Shatsky conjugate is consumed by 70 Ma by which time the Farallon underplates much of central USA, producing tectonic uplift associated with the Laramide Orogeny. (**c**) At 50 Ma the Farallon plate is removed from the base of overriding continental lithosphere resulting in widespread kimberlite eruptions. Black polygons indicate reconstructed large igneous provinces, grey polygons indicate inferred LIP conjugates, arrows indicate absolute plate velocity. Maps were generated using Cartopy^21^.

A second population of kimberlite eruptions occurred between 110 and 40 Ma while North America migrated westward during the opening of the North Atlantic Ocean. It has been proposed that the dehydration of hydrous minerals stored within the flat-subducting Farallon plate promoted magmatism and kimberlite generation approximately 1500 km from the nearest trench^45^, however, geodynamic models suggest that flat subduction inhibits arc magmatism as the release and convection of fluids from the slab are obstructed by the asthenospheric wedge^57^. From our reconstructions of slab dip, the average dip angle along the western margin of North America varies between 30 and 36$^\circ$ and the slab flux predicts the peaks and troughs in kimberlite eruption frequency between 110 and 40 Ma (Fig. 6c). Slab dip is spatially and temporally variable along North American subduction boundaries during the Laramide period, which has been attributed to the flat subduction of the Shatsky Rise conjugate on the northernmost section of the Farallon plate^40^. Its subduction predicts the distribution of magmatic and amagmatic zones in North America. From 95 to 60 Ma, the subduction of relatively young seafloor (5–50 Ma) combined with subduction of the buoyant conjugate Shatsky Rise leads to flat slab subduction beneath central USA^58^ (Fig. 7). The distribution of kimberlite eruptions during this period are focused in Canada and the south of North America on either side of the conjugate (Fig. 8). Abrupt changes in subduction angles could be accommodated by slab tears adjacent to the Arizona–New-Mexico magmatic belt^57^. It is likely that melts associated with the dehydration of recycled slab material in the mantle transition zone were delivered to the surface through subduction-induced return flow^47^. Removal of the flat Farallon slab from the base of overriding continental lithosphere at 50 Ma^59^ would further stimulate mantle return flow, triggering more widespread kimberlite eruptions which occur within the formerly amagmatic zone of central USA (Fig. 6c).

### A unifying mechanism for kimberlite eruptions?

The dichotomy of kimberlite eruption between African and North American populations is important because, while the mantle return flow mechanism is consistent, the stimulation of source regions in the mantle is different. In Africa, the mantle return flow associated with subducting slabs had likely stimulated upwellings along the edges of the LLSVP and invigorated a fertile mantle reservoir derived from sinking slab remnants left over from the assembly of Pangea, which explains the subduction signatures observed in this Cretaceous kimberlite population. The second pulse of African kimberlite eruptions at 80–90 Ma is likely connected to an increase in plate velocity as Africa migrates over upwellings associated with the LLSVP^3^. Meanwhile, kimberlite eruptions in North America are driven by upper mantle return flow in regions adjacent to the flat subduction of the Shatsky Rise and within the region affected by the Laramide Orogeny following the removal of the flat slab beneath North America. Importantly, both kimberlite populations are linked to the rapid subduction of the Farallon plate at low angle beneath the Americas, which suggests that this slab plays an important role in driving upwelling-induced volcanism. Whether the the Farallon slab is imbued with a high enrichment of volatiles, such as H$_2$O which lowers the solidus temperature promoting partial melting and magma generation^45^, or if it stimulates preexisting fertile mantle reservoirs is unclear. Nevertheless, this study highlights the importance of subduction on the generation of kimberlites, and challenges previous conceptions that kimberlites are primarily generated by mantle plumes.

## Conclusions

The dip angle of subducting oceanic lithosphere is a key parameter which characterises mantle and continental dynamics at subduction zones. We propose a simple framework for predicting slab dip from the thickness of the down-going slab, the convergence rate, the rate of trench migration, the density and volatile enrichment of the slab. Applying this framework to plate reconstructions provides new insights into the dynamics of past subduction zones, the spatial distribution of arc volcanism through deep geological time, and the fate of subducted slabs. Using this predictive framework, we reconstruct the slab dip angle of subduction zone segments over the last 170 million years to help explain pulses in kimberlite eruptions. High subduction rates stimulate mantle return flow which promotes partial melting and magma generation. Kimberlite eruptions in Africa and North America are linked to the subduction of the Farallon plate beneath the Americas. In Africa, peaks in kimberlite eruptions exhibit a strong correlation with high slab flux during the initial stages of supercontinent breakup and high plate velocities (up to 6 cm/yr) as Africa migrates over the LLSVP. In North America, the subduction of the Shatsky Rise conjugate from 95–50 Ma results in flat subduction beneath central USA, associated with the Laramide Orogeny, which limits magmatism to the edges of the flat slab until its removal from the base of the lithosphere after 50 Ma. Our results highlight the important role of subduction angle in modulating horizontal slab flux and thereby the distribution and timing of volcanism. This helps to explain the dichotomy of kimberlite populations in Africa and North America and has important implications for the formation of ancient kimberlites in Australia, India, and South America which may be explained by reconstructions of slab dip through deep geological time.

## Methods

The plate model used in this paper was modified from a recently-published model^15^ as follows. Subduction zones to which no motions assigned were initially stationary through time; these were assigned new motions relative to the global plate circuit to model moderate trench retreat while still remaining consistent with tomographic constraints. The Orcas plate was split into two separate plates at 170–130 Ma, consistent with its configuration after 130 Ma, in order to accommodate divergence at the plate boundaries. The Caribbean plate was also split by a new back-arc spreading centre at 140–120 Ma, to allow for the formation of the Caribbean large igneous province at a spreading ridge^51^. Finally, the absolute plate motion model was constrained using an iterative optimisation workflow^24^. A ZIP archive containing plate reconstruction files for use in GPlates is available on Zenodo (doi.org/10.5281/zenodo.5769002).

## Supplementary Information

Supplementary Information 1.

Supplementary Information 2.

## Data Availability

The datasets generated and/or analysed during the current study are available in the Zenodo repository, https://doi.org/10.5281/zenodo.5831990. A series of Jupyter notebooks containing a Python workflow to calculate slab dip using a plate reconstruction model is also available at Zenodo via https://doi.org/10.5281/zenodo.5831990 and GitHub (https://github.com/brmather/Slab-Dip). The plate reconstruction software, GPlates and pyGPlates, are freely available from www.gplates.org/download.
